# Immune Related Genes Underpin the Evolution of Adaptive Immunity in Jawless Vertebrates

**DOI:** 10.2174/138920212799860670

**Published:** 2012-04

**Authors:** Nathanael McCurley, Masayuki Hirano, Sabyasachi Das, Max D Cooper

**Affiliations:** Emory Vaccine Center and Department of Pathology and Laboratory Medicine, Emory University, Atlanta, Georgia, USA

**Keywords:** Adaptive immunity, Antigen receptor, Evolution, Jawless vertebrate, Leucine-rich repeat, Phylogeny, Somatic diversification, Variable Lymphocyte Receptor.

## Abstract

The study of immune related genes in lampreys and hagfish provides a unique perspective on the evolutionary genetic underpinnings of adaptive immunity and the evolution of vertebrate genomes. Separated from their jawed cousins at the stem of the vertebrate lineage, these jawless vertebrates have many of the gene families and gene regulatory networks associated with the defining morphological and physiological features of vertebrates. These include genes vital for innate immunity, inflammation, wound healing, protein degradation, and the development, signaling and trafficking of lymphocytes. Jawless vertebrates recognize antigen by using leucine-rich repeat (LRR) based variable lymphocyte receptors (VLRs), which are very different from the immunoglobulin (Ig) based T cell receptor (TCR) and B cell receptor (BCR) used for antigen recognition by jawed vertebrates. The somatically constructed VLR genes are expressed in monoallelic fashion by T-like and B-like lymphocytes. Jawless and jawed vertebrates thus share many of the genes that provide the molecular infrastructure and physiological context for adaptive immune responses, yet use entirely different genes and mechanisms of combinatorial assembly to generate diverse repertoires of antigen recognition receptors.

## 1 INTRODUCTION

**Fig. (1). Adaptive immunity throughout vertebrate phylogeny.**
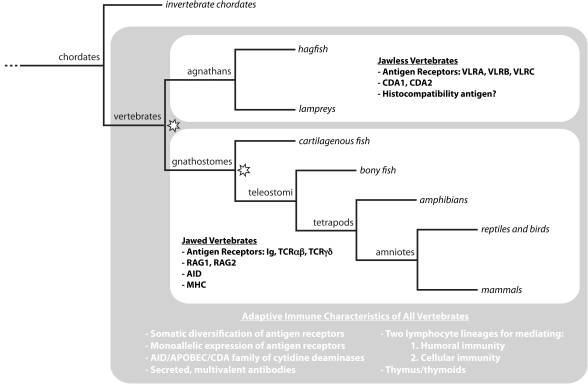
Vertebrates employ an adaptive strategy for immune defense that relies upon several universal characteristics. Jawless (agnathan) and jawed (gnathostome) vertebrate lineages differ, however, in several fundamental elements of adaptive immunity. Agnathans recognize antigens with VLRs constructed of LRR modules; CDA1 and CDA2 have been posited as critical mediators of VLR somatic diversification. Gnathostomes construct Ig and TCR antigen receptors from IgSF domains; in this lineage RAG1 and RAG2 are vital for gene assembly and AID for somatic hypermutation, class switching, and gene conversion. Two whole-genome duplications have been proposed during vertebrate evolution (indicated by stars).

All metazoans utilize germline-encoded pattern recognition receptors for detection of pathogens and initiation of innate immune responses [1, 2]. In addition, vertebrates can mount adaptive immune responses by recognizing specific antigens with somatically diversified antigen receptors that are expressed by activatable lymphocytes [3]. Although adaptive immunity is characteristic of all vertebrates, two very different molecular strategies for antigen recognition have evolved (Fig. **1**). In gnathostomes (jawed vertebrates), T and B lymphocytes use the somatically diversified T cell receptor (TCR) and immunoglobulin (Ig) genes, respectively, for adaptive immune responses. During their development in the thymus and hematopoietic tissues, respectively, T and B lymphocytes use V(D)J recombination to generate diverse repertoires of their Ig-based antigen receptors that are capable of recognizing >10^14^ potential antigens. The genes encoding the key recognition receptors of this adaptive immune system, Ig, TCR and major histocompatibility complex (MHC) genes, are present in all jawed vertebrates, yet none of them have been found in agnathans (jawless vertebrates). Instead, the two extant jawless vertebrates, lampreys and hagfish, have leucine-rich-repeat (LRR) genes in pieces that can be somatically assembled to encode variable lymphocyte receptors (VLRs) that are used for antigen recognition in adaptive immune responses [4-6]. Three VLR genes (*VLRA*, *VLRB* and *VLRC*) have been identified in lampreys and these are expressed in monogenic and monoallelic fashion by discrete populations of lymphocytes [7-9]. *VLRA* is expressed on the surface of T-like lymphocytes. *VLRB* is expressed on the surface of B-like lymphocytes, which respond to antigens by differentiation into plasma cells that secrete VLRB as a multivalent protein. The recently identified *VLRC* is expressed by yet another lymphocyte lineage. *VLRA* expressing lymphocytes develop in a thymus-like tissue (termed thymoid) at the tips of the gill filaments, while *VLRB* expressing lymphocytes develop in hematopoietic tissues [10]. Reciprocal expressions of cytokines and chemokines and their receptors imply communication between these lymphocytes for productive immune responses [8]. 

Agnathans lack* RAG1* and *RAG2* genes and recombination signal sequences for the VLR gene segments, whereas these are used by gnathostomes for V(D)J-mediated antigen receptor gene assembly. However, two AID-APOBEC family member genes, *CDA1* and *CDA2*, have been found in lamprey, and they are postulated to catalyze the assembly of VLR genes by a gene conversion-like mechanism that results in a potential antigen receptor repertoire of >10^14^ antigen specificities [7, 11]. Recent studies demonstrate the uniqueness of the jawless vertebrate immune system and also highlight underlying genetic elements and developmental processes that provide the framework for vertebrate adaptive immune systems. In this brief review, we consider some of the genetic attributes of vertebrates that are relevant to the evolution of adaptive immune competence, genes that facilitate immune function, and the unique features of the adaptive immune system in jawless vertebrates.

## 2 GENERAL COMPARATIVE GENETIC CONSIDERATIONS

Organisms across the animal phyla allocate a large proportion of their genome to immune defense [12]. Evolution of the vertebrate lineage resulted in additional phenotypic and genetic innovations that include novel mechanisms of immune defense [13]. The blossoming of new genes and genetic mechanisms in vertebrates is often considered in the context of the Ohno hypothesis, which posited two rounds of genome-wide duplications that permitted innovation by making available an excess of genes, modifications of which could be selected for novel functions [14]. Comparisons of vertebrate gene families with those of more basal invertebrate chordates have confirmed that the vertebrate lineage underwent two rounds of genome duplication [15, 16]. It has been proposed that the first of these occurred in the common vertebrate ancestor and the second in the jawed vertebrate lineage after its divergence from jawless vertebrates (Fig. **1**), although the timing of the genome duplications is still in doubt [17, 18]. The sequencing of the sea lamprey genome is currently underway and its availability should facilitate resolution of this issue. The currently available information indicates that jawed and jawless vertebrates share a remarkable number of novel gene families and gene regulatory mechanisms [7, 19-22]. 

An important distinction between vertebrates and invertebrates, including the most basal chordates, amphioxus and tunicates, is a variation in the mechanism of gene regulation by CpG methylation [23] which gives rise to noteworthy immunological considerations. All vertebrates methylate the majority of CpG sequences in their genomes, leaving short unmethylated CpG islands that facilitate differential gene expression. Conversely, invertebrate genomes have a mosaic pattern of CpG methylation with only 10-15% of CpG sequences being in the methylated state [24, 25]. This represents a fundamental change in that vertebrates rely heavily upon this epigenetic mark for gene silencing, controlling locus accessibility, and for preventing spurious gene expression. Early in ontogeny, vertebrate genomes are epigenetically reprogrammed by genome-wide erasure of CpG methylation patterns [26]. Surprisingly, activation-induced cytidine deaminase (AID) is a key mediator of the demethylation. The *aicda* gene (called *AID* here), which belongs to the AID/APOBEC family of vertebrate cytidine deaminase genes, was originally identified for its role in antigen receptor diversification in jawed vertebrates through somatic hypermutation, class switching, and Ig gene conversion. [27-29]. The phylogenetic appearance of the AID/APOBEC family in the agnathans coincides with the emergence of both adaptive immunity and global CpG demethylation, leading to the question of which of these functions came first and whether one facilitated the others. 

Secondly, programmed gene rearrangements outside of the context of meiosis are rare among invertebrates. In vertebrates, these events require that the affected locus be demethylated and transcriptionally active. It is therefore conceivable that global methylation serves in part to protect against adventitious rearrangements and mutation in animals that possess the AID/APOBEC family of genes. AID cuts both ways, however, since it also facilitates CpG demethylation in the correct developmental context.

Other gene regulatory factors emerged in the vertebrate lineage. A cadre of recently identified microRNAs may have originated in the common vertebrate ancestor [20, 30]. These microRNAs and their tissue-specific expression patterns are conserved in both jawed and jawless vertebrates, suggesting that their roles in development function and are retained. In addition to discrete regulatory elements, vertebrates also have evolved gene regulatory networks that facilitate both physiological and anatomical innovations, as has been most thoroughly investigated in neural crest development [31, 32].

Two notable distinctions have been elucidated for the agnathan and gnathostome genomes. First, in a process independent of antigen receptor gene assembly, lampreys irreversibly excise large portions of their genomes during development and hagfish expunge full chromosomes [22]. Agnathans are the only vertebrates in which such programmed loss is known to occur and the mechanisms and functions of this phenomenon are still incompletely understood. Both coding and non-coding sequences are lost in this process and the loss may not be uniform across different tissues [33]. If genes related to immune function are lost, this process would be relevant to the development of immune competence. 

Another fundamental distinction lies in the genes directly involved in the generation and maintenance of adaptive immune responses [6]. While jawed vertebrates construct antigen receptors from Ig superfamily (IgSF) genes and use a transposase mechanism of gene segment recombination mediated by the *RAG1* and *RAG2* genes [34, 35], the antigen receptors of jawless vertebrates are constructed of LRR modules that are assembled in a process resembling gene conversion. Two cytidine deaminase (CDA) genes of the AID/APOBEC family, *CDA1* and *CDA2*, have been implicated in the process of VLR gene conversion. This constitutes a significant variation in the genome biology of these two vertebrate lineages, not only because of the considerable size (and number) of loci involved, but because both mechanisms for antigen receptor gene assembly have enormous potential for compromising genome integrity by generating double strand breaks in the process.

## 3 SHARED IMMUNE RELATED GENES

**Fig. (2). Adaptive immunity in the context of conserved immune related genes.**
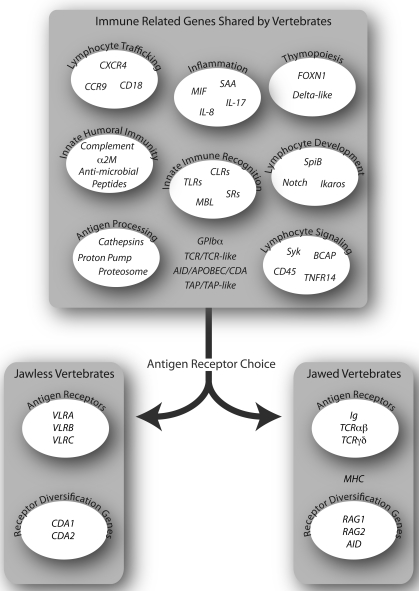
The adaptive immune systems of jawless and jawed vertebrates differ fundamentally in the antigen receptors used and in the mechanisms for receptor diversification. In both lineages, however, common sets of immune related genes in different functional categories facilitate the development and operation of adaptive immunity.

Many immune related genes (IRGs) that are not directly involved in somatic diversification of antigen receptor genes are shared amongst the vertebrates, quite a few of which are found in invertebrates (Fig. **2**). IRGs are used to mediate a broad range of immune functions, such as innate immune recognition (toll-like receptors, scavenger receptors, C-type lectin receptors) [8, 21, 36-38], inflammation (macrophage migration inhibitory factor (MIF), interleukin 8 (IL-8), IL-17, serum amyloid A (SAA)) [8, 39], leukocyte trafficking (CCR9, CXCR4) [8], wound healing (coagulation factors) [40], soluble humoral effectors (complement component C3, Factor B, MASP, MBL, C1q; alpha-2 macroglobulin (α2M)) [40, 41], phagocytosis and lysosomal degradation (Rho-family GTPases, PIP kinases, talin, cathepsins, vacuolar H^+^-ATPase) [40, 42-44]. Other IRGs provide direct support for the development and function of clonally diverse lymphocytes which mediate adaptive immunity in agnathans and gnathostomes [8, 10, 45-47]. These include genes for lymphocyte development (Notch, GATA2/3, Ikaros, SpiB), other lymphocyte transcription factors (c-Rel, aryl hydrocarbon receptor, BCL11b), lymphocyte signaling (Syk, BCAP), and genes expressed by thymic epithelial cells (FOXN1, delta-like B (DLL-B)). The abundance of shared IRGs illustrates that adaptive immunity relies upon a common basal set of genes and gene regulatory networks. 

Several of the genes which are important for adaptive immunity in gnathostomes are linked in the MHC locus. A “proto-MHC” locus has been identified in amphioxus which lacks orthologs of the gnathostome MHC class I and II genes, but contains at least nine anchor genes that define the MHC locus [17, 48]. Importantly, this proto-MHC contains several IRGs, including the C3 family of complement components, proteosomal subunits, and *Notch*, a gene important in lymphocyte development. In keeping with the Ohno hypothesis of two rounds of genome duplication, there is one proto-MHC locus in amphioxus and four paralogous loci in jawed vertebrates, one of which is the MHC locus [18]. The sea lamprey, for which the most genetic information is available among the agnathans, has orthologs of genes in the amphioxus proto-MHC and orthologs of gnathostome MHC-linked IRGs, including a TAP-like gene [49] and a family of cathepsins [40, 50]. Whether these agnathan genes are organized in a locus (or loci) orthologous to MHC (or proto-MHC) is unknown, because of the fragmentary information on the lamprey and hagfish genomes. The future availability of these genomes will facilitate investigation of MHC orthologs and the timing of the two rounds of genome-wide duplication that occurred in the vertebrate lineage [15]. 

## 4 EMERGENCE OF ALTERNATIVE ADAPTIVE IMMUNE SYSTEMS IN VERTEBRATES

The use of somatic chromosomal recombination in antigen receptor loci to generate a diverse receptor repertoire is a novel feature shared by all vertebrates. This unique feature is universally maintained despite the fact that the antigen receptor genes and the mechanisms of somatic gene assembly differ fundamentally between agnathans and gnathostomes [6]. 

Although many genes that provide a context for adaptive immunity are common to both vertebrate lineages, different ancestral genes were chosen by the two lineages for somatic diversification. Candidates for the ancestral genes of the IgSF-based TCR and Ig genes have been identified in lampreys [51], hagfish [52], and amphioxus [53] on the basis of their position of extracellular Ig domains that have V- and J-like sequences, athough none of these candidates are somatically diversified. *GPIbα* (*CD42b*), a transmembrane LRR protein on platelets that binds von Willebrand’s clotting factor, has sequence similarity with the VLRs. It is the only other LRR protein that has an insert in its LRRCT, one that is invariant however. *GPIbα*, which is unique to vertebrates, has therefore been proposed as ancestral to the agnathan VLR genes [7]. Thus the raw genetic material for both types of antigen receptors was likely present in the common vertebrate ancestor. The distinction between the jawed and jawless vertebrate adaptive immune system came with the choice of antigen receptor and the mechanism of somatic diversification (Fig. **2**) [54]. 

## 5 GENE STRUCTURE OF VLRS AND THE ARCHITECTURE OF VLR LOCI

**Fig. (3). VLR gene assembly in lamprey lymphocytes.**
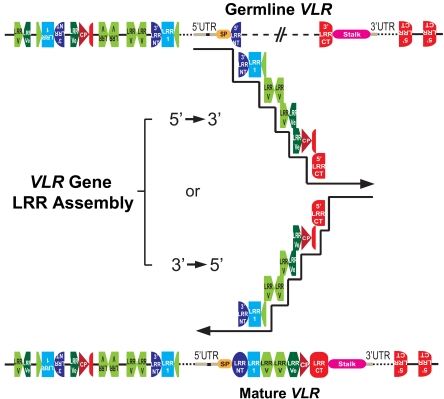
The incomplete germline VLR genes are flanked by hundreds of LRR cassettes. The noncoding intervening sequence of germline VLR is replaced by LRR fragments that are sequentially copied from the flanking LRR cassettes. Mature complete VLR genes are assembled from either the LRRNT or LRRCT end in a stepwise fashion that is directed by short sequence homology between the donor and recipient LRR sequences. This figure was modified from [6].

The details of jawed vertebrate antigen receptor loci and mechanisms of somatic rearrangement have been discussed in detail elsewhere [18, 35]. The antigen receptors of agnathans are encoded by three gene loci named *VLRA*, *VLRB*, and *VLRC* [4, 7, 9, 55]. Since *VLRC* was only recently identified in lampreys and has not been identified in hagfish, our discussion focuses on *VLRA* and *VLRB*. In the germline, each VLR gene is found in incomplete form containing the 5’ UTR (harboring the only intron of these genes), a portion of the 5’ coding region of the gene (5’ LRRNT), a non-coding intervening sequence, and finally the 3’ coding region of the gene (3’ LRRCT and stalk) (Fig. **3**). In the case of lamprey *VLRB*, there are two intervening sequences separated by a coding region containing the 5’ end of the LRRCT. In sea lamprey this incomplete gene spans approximately 0.9 kb for *VLRA*, 14 kb for *VLRB*, and 1.2 kb for *VLRC*. 

Flanking the incomplete VLR genes, there are many modular LRR gene sequences located upstream and downstream of the germline genes. Each of these flanking cassettes contains sequences for one, two, or three LRR modules, or fractions thereof; they can be oriented in either the forward or reverse direction with respect to the incomplete VLR gene. The sizes of the VLR loci containing the incomplete VLR gene and hundreds of gene-specific VLR cassettes are approximately 2.2 megabases for *VLRA* and 2.1 megabases for *VLRB* [7]. The cassettes are selected randomly as templates for the assembly of functional VLR genes using a mechanism similar to gene conversion (see below). *VLRA* and *VLRB* do not share cassettes for gene assembly, and it is currently unknown if any of the lamprey VLR loci are linked. Utilizing fluorescent *in situ* hybridization probes for *VLRA* and *VLRB* genes in hagfish, the two loci were shown to be linked, although far apart on the chromosome [56].

Once fully assembled, the mature VLR genes encode a signal peptide, a capping 27-38 residue N-terminal LRR (LRRNT), an 18-residue LRR (LRR1), one to eight 24-residue LRRs (LRRV), a 24-residue end LRR (LRRVe), one 13-residue connecting peptide (CP), a capping 48-65 residue C-terminal LRR (LRRCT), and a conserved C-terminus containing a stalk region (Fig. **3**). The LRR-containing portions of the VLR proteins fold into the curved solenoid structure that is typical of LRR proteins, the concave surface of which mediates antigen binding [57-61]. A highly variable insert in the LRRCT domain of assembled *VLRA* and *VLRB* genes encodes an extended loop structure that is important for antigen binding [5]. Only one other LRRCT loop-containing protein has been found to have a comparable LRRCT insert *GPIbα*, and it is invariant. The *GPIbα* gene is present in all vertebrates, but not invertebrates, and may represent the ancestral gene for the VLRs [7]. The invariant stalk region of VLRs is rich in threonine and proline and is postulated to form an extended, glycosylated scaffold with flexibility for orienting of the antigen-binding domain [58]. The sequence of the stalk region differs between VLR genes, thus allowing the development of antibodies specific for each VLR type [8]. 

VLR gene assembly occurs by monogenic rearrangement of VLRs in developing lymphocytes. The lymphocytes that rearrange *VLRA* do not rearrange *VLRB*, and *vice-versa* [8]. Regulation of transcription likely plays an important role in the monogenic choice, since transcription is coincident with the VLR gene assembly. Cells that achieve productive *VLRA* assembly do not transcribe *VLRB*, and vice-versa [8, 62]. Consistent with the considerable distance between the two loci, the *VLRA* and *VLRB* cassettes are not shared as potential donors for assembly of the mature *VLRA* and *VLRB* genes, and are rather used exclusively of one another [7]. Given a gene conversion-like mechanism of VLR rearrangement, cassette sharing is theoretically possible. Since this does not occur, it suggests that the entire *VLRB* locus is transcriptionally silenced during rearrangement of *VLRA*, and *vice versa*. The mechanisms of transcriptional regulation of VLR expression are still under investigation, but the available data imply that VLR locus accessibility during VLR assembly may be under epigenetic control. 

In addition to monogenic combinatorial assembly, VLRs also exhibit monoallelic assembly. Interestingly, both VLR alleles are transcribed although only one undergoes productive assembly [4, 11, 55, 62, 63]. In jawed vertebrates, a similar phenomenon has been observed for the *Igκ* and *TCR Vβ* genes during their rearrangement. These observations imply that allelic exclusion in both types of adaptive immune systems is mediated by mechanisms other than mere locus accessibility [64, 65]. Occasional cells have been observed to have one productively assembled VLR allele with non-productive assembly of the other, thereby suggesting a quality control mechanism that allows assembly of the second allele only when the first is non-productive. No instances have been identified in which both alleles were productively rearranged. The one cell-one receptor phenomenon is essential for maintenance of the specificity and memory in adaptive immune responses. Study of VLR gene assembly therefore offers a useful comparative approach for unraveling the mechanisms of allelic exclusion.

## 6 MECHANISM OF VLR ASSEMBLY

The somatic diversification of antigen receptor genes is a basic feature of adaptive immunity. Jawed vertebrates generate somatic diversity of antigen receptor genes by several means which are briefly considered here for comparison with jawless vertebrates. Diversity is primarily introduced by random combinatorial assembly of V, D, and J gene segments into functional antigen receptor genes; the rearrangement process is initiated by the recombination activating gene products, RAG1 and RAG2 [66, 67]. Junctional diversity is superimposed by generating random sequences at the junction of V, D, and J segments through nucleotide trimming at the junction, the addition of palindromic nucleotides, and the random addition of non-templated nucleotides by the enzyme terminal deoxynucleotidyltransferase or TdT [68, 69]. Together these processes result in potential antigen receptor repertoires of >10^14^. In addition, following antigen encounter the Ig variable regions of B cells undergo somatic hypermutation as a further means of diversification; this third level of diversity is mediated by AID activity in jawed vertebrates [70]. 

Somatic diversification of the agnathan antigen receptor genes occurs in a similar, yet different fashion. Hundreds of LRR cassettes that flank an incomplete VLR gene are available for use as donor sequences during VLR gene assembly. Sequences from randomly selected donor LRR cassettes are sequentially incorporated into a VLR gene to achieve combinatorial diversity [4, 11]. The donor LRR cassettes serve as templates, and are not rearranged during the copying process; short stretches of nucleotide similarity between donor and recipient sequences may facilitate their temporary association during the copying process. Since multiple germline LRR cassettes often contribute to a single mature LRR module, this patchwork assembly process increases the sequence diversity of *VLRA* and *VLRB *genes to yield a potential repertoire >10^14^ for each VLR gene, which is comparable to the potential diversity of the TCR and Ig genes [7, 11, 63].

The mechanistic details of VLR gene assembly are unknown, but the process resembles gene conversion. Two AID orthologs have been identified in lampreys, CDA1 and CDA2, and these enzymes are posited to be key elements in the VLR assembly process [7]. *CDA1* is expressed exclusively by VLRA+ lymphocytes during their initial development in the lamprey thymoid, whereas *CDA2* expression is restricted to the VLRB+ lymphocyte lineage, primarily by cells located in hematopoietic tissues [8, 10]. In support of their presumed functional role in VLR gene assembly, CDA1 has been shown to have cytidine deaminase activity and to be capable of inducing both point mutations in *E. coli* and intragenic recombination in yeast [7]. In the process of VLR gene assembly double strand breaks (DSB) are created that must be repaired [71]. The programmed introduction of DSB increases the risk of chromosomal translocations and aberrant gene expression that may predispose the development of malignancies. The genes for DSB repair are very ancient and likely play vital roles in agnathan antigen receptor assembly as in gnathostomes [72]. Further investigations of VLR gene rearrangement will thus provide an opportunity to examine the mechanisms of maintenance of genome integrity from an alternative perspective.

In jawed vertebrates, AID expression induces somatic hypermutation of the Ig variable region to amplify the repertoire diversity of B cells. The expression of CDA2 by lamprey VLRB+ lymphocytes suggests the possibility of tertiary diversification *via *somatic hypermutation. While there is currently no experimental evidence that this occurs in lamprey lymphocytes, it will be particularly interesting to investigate the possibility that affinity maturation of VLRB antibodies occurs during antigen reencounter of the agnathan B-like cells.

## 7 FUNCTIONAL DIVERSITY OF VLRA, VLRB, AND VLRC

Microorganisms can invade and grow within intracellular or extracellular compartments of multicellular organisms. The gnathostome T and B lymphocytes have overlapping and complementary roles in monitoring these two niches [54]. Whereas B cells recognize extracellular antigens and secrete soluble antibodies that facilitate agglutination, opsonization, neutralization, and clearance of the antigens, T cells use their antigen receptors to recognize MHC molecules bearing processed peptide antigens derived from either intracellular or extracellular sources. 

The first agnathan VLR gene locus to be discovered (*VLRB*) was shown to generate a vast repertoire of potential antigen receptor specificities, which could also be secreted as antibodies [63]. Highly specific VLRB antibodies are produced in response to immunization with bacteria, viruses, fungi, and xenogeneic vertebrate cells [8, 73]. The VLRB antibody responses are long-lived and the antibody levels increase by about 20-fold after booster immunization as an indication of antigen-specific memory. VLRB antibodies are secreted as multimers consisting of 8-10 VLRB chains, which accounts for their high avidity and effective agglutination function [58]. The VLRB-producing lymphocytes thus may provide antigen-specific humoral immunity against extracellular pathogens. 

The second VLR locus to be identified (*VLRA*) is expressed by a separate lineage of lymphocytes that resemble the T lineage cells in jawed vertebrates [8]. While the VLRA receptor repertoire is comparable to that of the VLRB repertoire, VLRA expression is restricted to the cell surface. VLRA+ cells respond to stimulation by antigens and T cell mitogens by proliferation and upregulation of cytokines, such as IL-17 and MIF. However, binding to native antigens has not been shown for VLRA+ cells, and it is possible that the VLRA+ cells recognize processed antigens. By way of further analogy to T cells of jawed vertebrates, *VLRA* assembly appears to occur in the thymoid, a thymus-equivalent lymphoepithelial tissue in the gill region of lamprey larvae. 

The VLRA+ and VLRB+ cells show a reciprocal expression of cytokines and chemokines that implies their functional interaction. VLRA+ cells express IL-17 and the IL-8 receptor, while VLRB+ cells express the IL-17 receptor and IL-8 [8]. Upon immunization, VRLA+ cells upregulate their expression of IL-17 and VLRB+ cells upregulate IL-8. This illustrates a remarkable example of convergent evolution, wherein two independent strategies are used for generating diverse antigen receptor repertoires for two functionally intertwined lymphocyte lineages to provide immune surveillance of different niches and to cooperate in protective immunity. 

A third VLR gene locus, *VLRC*, has recently been discovered in lampreys [9]. *VLRC* gene assembly and expression occurs in an independent lineage of lymphocytes. *VLRC* gene sequences are most closely related to *VLRA,* but the *VLRC* locus has a more limited diversity of LRRCT cassettes. Furthermore, the *VLRC *LRRCT lacks a sequence encoding for a protruding loop, whereas variable LRRCT inserts that are found in *VLRA* and *VLRB* are key antigen binding elements. This feature suggests that VLRC may bind antigens differently than VLRA or VLRB. It has been proposed that the VLRA+ and VLRC+ lymphocyte lineages are lamprey counterparts to the αβ and γδ T cell lineages in jawed vertebrates. 

## 8 CONCLUSIONS

Immune defense promotes genetic continuity from one generation of organisms to the next by providing a check against assault on genomic integrity. Invertebrates use germline-encoded pattern recognition receptors for protective immune responses. The fact that all of the extant vertebrates have apparently evolved adaptive immune systems argues forcefully for the protective benefits of an adaptive immune system. Both of the vertebrate adaptive immune systems rely upon the introduction of chromosomal DSBs to facilitate somatic diversification of their antigen receptors on lymphocytes, thereby regularly risking genome integrity for the purpose of preserving it. The potential adverse consequences are evident in the numerous lymphomas and leukemias that have been attributed to genome instability consequent to V(D)J recombination [74-76] and AID activity [77-79]. Although agnathan antigen receptor genes and somatic diversification mechanisms differ from those of gnathostomes, a similar level of antigen receptor diversity is achieved and similar risks for malignancy may also be expected.
